# In vitro–in vivo correlation from lactide-co-glycolide polymeric dosage forms

**DOI:** 10.1007/s40204-014-0029-4

**Published:** 2014-12-02

**Authors:** Susan D’Souza, Jabar A. Faraj, Stefano Giovagnoli, Patrick P. DeLuca

**Affiliations:** 1 University of Kentucky College of Pharmacy, Lexington, KY 40536 USA; 2 Present Address: Sunovion Pharmaceuticals Inc, Marlborough, MA 01752 USA; 3 Present Address: Evonik Inc, 750 Lakeshore Parkway, Birmingham, AL 35211 USA; 4 Present Address: Department of Chemistry and Technology of Drugs, Università degli Studi di Perugia, Via del Liceo 1, 06123 Perugia, Italy

**Keywords:** Risperidone, PLGA microspheres, In vitro–in vivo correlation, Level A, Modified dialysis method, Nelson–Wagner, Fractional AUC

## Abstract

The objective of this study was to compare the in vitro behavior of four long-acting subcutaneous risperidone formulations with in vivo performance, with the intent of establishing an IVIVC. Two copolymers of PLGA (50:50 and 75:25) were used to prepare four microsphere formulations of risperidone, an atypical antipsychotic. In vitro behavior was assessed at the physiological temperature (37 °C) using the ‘modified dialysis’ technique. The in vitro release profile demonstrated rank order behavior with *Formulations A and B*, prepared using the 50:50 copolymer, exhibiting rapid drug release, while *Formulations C and D*, prepared using 75:25 PLGA, released drug in a slower manner. In vivo profiles were obtained by two approaches, i.e., deconvolution using the Nelson–Wagner equation (the FDA recommended approach) and using fractional AUC. With both in vivo approaches, the 50:50 PLGA preparations released drug faster than the 75:25 PLGA microspheres, exhibiting the same rank order observed in vitro. Additionally, profiles for the four formulations obtained using the deconvolution approach were nearly superimposable with fractional AUC, implying that the latter procedure could be used as a substitute for the Nelson–Wagner method. A comparison of drug release profiles for the four formulations revealed that in three of the four formulations, in vivo release was slightly faster than that in vitro, but the results were not statistically significant (*P* > 0.0001). An excellent linear correlation (*R*^2^ values between 0.97 and 0.99) was obtained when % in vitro release for each formulation was compared with its corresponding in vivo release profile, obtained by using fraction absorbed (Nelson–Wagner method) or fractional AUC. In summary, using the four formulations that exhibited different release rates, a Level A IVIVC was established using the FDA-recommended deconvolution method and fractional AUC approach. The excellent relationship between in vitro drug release and the amount of drug absorbed in vivo in this study was corroborated by the nearly 1:1 correlation (*R*^2^ greater than 0.97) between in vitro release and in vivo performance. Thus, the results of the current study suggest that proper selection of an in vitro method to assess drug release from long-acting injectables will aid in obtaining a Level A IVIVC.

## Background

The delivery of therapeutic agents using controlled release polymers has been an area of research that has witnessed extensive growth. Indeed, the nature and properties of these polymers lend themselves well to the design and development of complex dosage forms, whether for administration via the oral or non-oral route. One such polymer that has consistently demonstrated success with controlled release injectables is the PLGA (polylactide-co-glycolide) copolymer (Schrier and DeLuca 1999; Hora et al. 1990; Mehta et al. 1994; Capan et al. 1999). The PLGA polymer is biodegradable, biocompatible and has been approved for human use by the United States Food and Drug Administration (US FDA) as surgical sutures, implantable devices and drug delivery systems (D’Souza et al. 2013c; Middleton and Tipton 2000; He et al. 2011; Wei et al. 2004). Formulation of an injectable with a versatile polymer like PLGA provides significant benefits. For instance, PLGA-based formulations such as biodegradable microspheres are able to bypass the GI tract and allow enhanced bioavailability of molecules with a short half-life. e.g., peptides and proteins. Other advantages include reduced frequency of dosing, improved patient compliance, maintenance of consistent blood levels and less systemic side effects due to controlled delivery of the therapeutic agent upon intramuscular or subcutaneous administration (D’Souza et al. 2013a; Xuan et al. 2013; Shmueli et al. 2013; Kwak et al. 2010). Thus, the long history of safety and advantages of the PLGA polymer have resulted in several commercialized formulations and billions of dollars in revenue (Chaubal 2002).

Several physical and chemical polymer properties regulate drug release from the PLGA polymer. These include molecular weight, copolymer composition, crystallinity and hydrophilicity (DeLuca et al. 1993; Park et al. 2007; Xuan et al. 2013; Sun et al. 2009). A careful selection of polymer and dosage form properties ensures customized drug release for varying duration of action, ranging from days to several months, as evidenced by numerous reports (Ertl et al. 1999; Woo et al. 2001). Hence, monitoring drug release from PLGA-based formulations, both in vivo and in vitro, routinely requires extended study periods. In vivo measurements of drug release from injectable dosage forms, though preferred, entail considerable time to plan and conduct, are expensive and labor-intensive. In contrast, in vitro studies, a surrogate for in vivo assessments, are much simpler to perform and provide extensive insight into the rate and mechanism of drug release (D’Souza et al. 2014a; Washington 1990). Therefore, investigations of in vitro–in vivo correlations (IVIVC) between in vitro drug release and in vivo bioavailability are progressively becoming central to the development of extended-release products (Uppoor 2001; Siewert et al. 2003; Martinez et al. 2008). However, when compared with conventional dosage forms, literature on IVIVC with extended-release injectables continues to remain sparse.

For an IVIVC, it is critical to select the appropriate in vitro conditions such that the in vivo environment is mimicked as closely as possible without any change in the release mechanism(s). While in vitro evaluation of dosage forms like oral tablets or capsules is straightforward because compendial apparatus can be utilized, measurement of drug release from non-oral dosage forms like injectable microspheres is not as simple. Lack of a compendial apparatus has led to a proliferation of apparatus that are used to assess drug release (D’Souza and DeLuca 2006). For this reason, several methods have been developed to study drug release from injectable microspheres.

A literature survey reveals that in vitro drug release methods for injectable dosage forms such as PLGA microspheres fall into three categories. The most popular ‘sample and separate’ or ‘tube’ method involves introduction of the injectable dosage form into a container containing release media, and drug release is assessed over time by filtration [analysis of the filtrate (Yen et al. 2001)] or centrifugation [analysis of the supernatant (Park et al. 1998) or remaining drug (Blanco-Prieto et al. 2004)]. The ‘tube’ method, though simple to set up and use, has significant drawbacks. Lower drug release rates have been reported and are a consequence of aggregation of microspheres in the release media (Bain et al. 1999). Sampling and buffer replacement during the later stages of drug release are quite cumbersome due to the formation of small-sized particles (by-products of polymer degradation) causing filter clogging. Further, sink conditions are difficult to maintain due to challenges involved with partial or total buffer replacement of the release media for the extended duration of the in vitro release study. Nevertheless, IVIVCs with biodegradable microspheres have been attempted using the ‘sample and separate’ in vitro method, with varying degree of success (Jiang et al. 2002; Morita et al. 2001; Heya et al. 1994).

The ‘continuous flow’ method (an adaptation of USP apparatus 4), is another technique that has been used to study in vitro drug release from injectable microspheres (Aubert-Pouessel et al. 2002). In the ‘continuous flow’ method, the release media is circulated or re-circulated (via a pump) through a column containing injectable microspheres. Sampling of the release media at pre-determined intervals allows for an evaluation of drug release over a period of time. Several types of pumps have been reported including syringe pumps (Aubert-Pouessel et al. 2002, 2004), HPLC pumps (Longo and Goldberg 1985) and peristaltic pumps (Wagenaar and Muller 1994) allowing for a wide range of flow rates as low as 5 μL/min to a high 200 L/h (D’Souza and DeLuca 2006). Though the setup of the ‘continuous flow’ method permits convenient sampling followed by buffer replacement, major shortcomings have been reported. Constant flow rates are difficult to achieve in the later stages of polymer degradation. Indeed, polymer breakdown causes filter clogging and has been reported to result in high pressure buildup leading to variable flow rates. Other disadvantages include cumbersome setup, static charge issues with glass beads and challenges with rapid buffer replacement (partial or total) (Rawat et al. 2011). These challenges make it difficult to establish an IVIVC using the ‘continuous flow’ method.

A third method utilizing the ‘dialysis’ principle has also been reported in literature (Kostanski and DeLuca 2000; D’Souza et al. 2014a). In this method, the injectable microspheres are placed into a dialyzer containing a small volume of release media which in turn is introduced into a container contain a larger volume of release media (outer bulk). As the polymer degrades and drug is released, it diffuses out of the dialyzer into the outer bulk, from where it is sampled. This physical separation of the injectable microspheres from the outer bulk media eliminates filter clogging issues reported with the ‘tube’ and ‘continuous flow’ methods. A key advantage of the ‘dialysis’ method is that it mimics in vivo conditions where the injectable microspheres are immobilized upon subcutaneous or intramuscular administration and surrounded by a stagnant layer causing slow diffusion of drug since sink conditions are not maintained (Nastruzzi et al. 1993). Setup issues with older systems such as dialysis bags (Diaz et al. 1999) have been eliminated with the introduction of commercially available dialyzers (Woo et al. 2001; D’Souza et al. 2014a). Of the three methods, partial or total buffer replacement of the outer bulk is straightforward, allowing sink conditions to be maintained throughout the duration of the in vitro study. The simplicity of this method lends itself well to an evaluation of in vitro release. Indeed, published results have confirmed the utility of this method in establishing an IVIVC with biodegradable microspheres (D’Souza et al. 2014b; Kostanski et al. 2000).

Given the advantages of the ‘dialysis’ method over the ‘sample and separate’ and ‘continuous flow’ methods, its suitability for use in the development of an IVIVC is obvious. Therefore, the objective of this study was to develop an IVIVC using the ‘dialysis’-based technique for previously developed PLGA-based microsphere preparations of risperidone (D’Souza et al. 2013b). Of the different ‘dialysis’-based techniques, the ‘modified dialysis’ method was selected to evaluate in vitro release. Further, an IVIVC was attempted using two approaches, deconvolution and fractional AUC.

## Materials and methods

### Materials

Risperidone was purchased from Cipla Ltd., India, and PLGA 50:50 (45 and 74 kDa) and 75:25 (54 and 65 kDa) from Boehringer Ingelheim (Ingelheim, Germany) and Alkermes (Cambridge, MA). All other chemicals were obtained commercially as analytical-grade reagents.

### Preparation of microspheres

Risperidone PLGA microspheres were prepared by a solvent extraction/evaporation method, as described previously (D’Souza et al. 2013b). Briefly, a solution of drug and polymer was injected into an aqueous continuous phase under stirring with a Silverson L4R mixer (Silverson machines, MA, USA) at a pre-determined speed. The solvents were removed by stirring for 2 h at 40 °C. The resulting microspheres were recovered by filtration, washed and freeze dried in unit vials along with the diluent. Briefly, the four formulations prepared were:

- 45 kDa PLGA, 50:50 lactide:glycolide (*Formulation A*),
- 74 kDa PLGA, 50:50 lactide:glycolide (*Formulation B*),
- 54 kDa PLGA, 75:25 lactide:glycolide (*Formulation C*) and
- 65 kDa PLGA, 75:25 lactide:glycolide (*Formulation D*).

### Drug content

Drug content and encapsulation efficiency were determined for all the formulations. Briefly, 5–10 mg of microspheres was dissolved in 10 mL of acetonitrile; 40 mL 0.1 M acetate buffer, pH 4.0, added and the solution gently mixed. The solution was filtered through a PTFE syringe filter prior to analysis by HPLC. The analysis was performed by injecting 50 μL samples in a HPLC C-18 column in gradient mode. The mobile phases were (A) 0.1 % TFA aqueous solution; (B) acetonitrile with 0.1 % TFA. The gradient method was: 80 % A, 20 % B to 50 % A, 50 % B over 12 min at a flow rate of 1.5 mL/min. Measurements were made in triplicate. Drug content for *Formulations A*–*D* was determined to be 25, 34, 34 and 33 %, respectively (D’Souza et al. 2013b).

### In vitro release

The in vitro study was performed in 0.1 M phosphate buffered saline, pH 7.4, containing 0.05 % Tween-80^®^ and 0.1 % sodium azide using a ‘modified dialysis’ method (Kostanski and DeLuca 2000; D’Souza et al. 2014b). Risperidone PLGA microspheres were accurately weighed and placed in a 7-mL dialysis tube (Tube-O-Dilalyzer^®^, MWCO 300,000 Da) filled with 5.0 mL of release media, which in turn was placed in a 50-mL tube containing 40 mL of the same release medium (outer bulk). The contents of the larger tube were continuously stirred with a magnetic stirrer. All tubes were incubated at 37 °C. At each time point 1.0 mL was removed from the 50-mL tube (outer bulk) and 1.0 mL of fresh buffer was added. Risperidone content was determined by HPLC.

### In vivo study

Male Sprague–Dawley rats (*n* = 6) weighing ~300 g were used to evaluate the in vivo performance of risperidone microspheres. The microspheres were injected subcutaneously at the back of the neck (20–40 mg/kg dose of risperidone/rat) after reconstitution in a suitable vehicle containing sodium carboxymethylcellulose, mannitol and Tween 80^®^. Blood samples were collected from the tail vein at specific time points and centrifuged in Microtainer^®^ tubes (Becton–Dickinson, Franklin Lakes, NJ) to collect the serum. Serum samples were frozen and stored at −20 °C until analysis by an outside laboratory, Medtox Laboratories, MN.

### Development of an IVIVC

Currently, there is no FDA guidance on establishing IVIVC for injectable dosage forms. However, using the FDA document for solid oral dosage forms, IVIVC relationships can be extrapolated to include non-solid oral delivery systems (FDA guidance for industry: extended-release oral dosage forms: development, evaluation and application of in vitro/in vivo correlations 1997). Per the guidance, an IVIVC can be categorized as follows (Uppoor 2001; D’Souza et al. 2014b):

1. Level A correlation is a point to point correlation between in vitro dissolution and in vivo absorption. That is, the in vitro dissolution profiles are typically superimposable with in vivo absorption curves or may be made superimposable by use of an appropriate scaling factor.
2. Level B correlation describes a relationship between summary parameters such as in vitro dissolution rate and in vivo absorption rate (e.g., mean dissolution time, MDT, vs mean residence time, MRT). By its definition, it is not a point to point correlation as several in vivo curves can produce a similar MRT value or mean in vitro dissolution curve.
3. Level C correlation is a single point comparison of the amount dissolved in vitro at a particular time (e.g., *T*_50 %_) and an in vivo pharmacokinetic parameter (e.g., area under the curve, AUC). It does not describe the nature of the in vivo release profile, which is an important aspect in the characterization of performance from extended-release drug products.

In the current study, an IVIVC for the four risperidone PLGA formulations was determined using two approaches.

- Nelson–Wagner approach: Of the FDA-recommended approaches for developing an IVIVC, the Nelson–Wagner technique was selected as it is suitable for use in drugs that follow a one-compartment pharmacokinetic model. The fraction absorbed (*F*_abs_) was determined from the plasma concentration–time data by deconvolution using the Nelson–Wagner method as described in Eq. 1 (Wagner and Nelson 1963).

1 $$ F_{\text{abs}} \left( t \right) \, = \, {{\left[ {C \, \left( t \right) \, + \, k_{\text{e}} \times {\text{ AUC}}_{(0 - t)} } \right]} \mathord{\left/ {\vphantom {{\left[ {C \, \left( t \right) \, + \, k_{\text{e}} \times {\text{ AUC}}_{(0 - t)} } \right]} {\left[ {k_{\text{e}} \times {\text{ AUC}}_{{(0 - { \inf })}} } \right]}}} \right. \kern-0pt} {\left[ {k_{\text{e}} \times {\text{ AUC}}_{{(0 - { \inf })}} } \right]}}. $$

With the Nelson–Wagner equation, the pharmacokinetic profile is deconvoluted to obtain the in vivo absorption as a function of time and is plotted alongside the in vitro release data to assess the superimposability of the two profiles. If the two curves are superimposable and a linear relationship is obtained, it suggests a strong correlation between in vivo and in vitro drug release.

b. Fractional AUC approach: The area under the curve (AUC) was calculated using the trapezoidal rule (Eq. 2)

2 $$ {\text{AUC}}\left( {t_{1} - t_{2} } \right) = \left[ {\left( {C_{1} + \, C_{2} } \right)/2} \right] \times \left( {t_{2} - t_{1} } \right). $$

The fractional AUC was determined by dividing cumulative AUC at time ‘t’ with cumulative AUC_(0−last)_, as described in previous publications (Woo et al. 2001; Chu et al. 2006; D’Souza et al. 2014b) and plotted along with the % drug released in vitro. In a manner similar to the Nelson–Wagner approach, the superimposability of the in vivo and in vitro drug release was compared.

## Results

### In vitro release

**Fig. 1:**
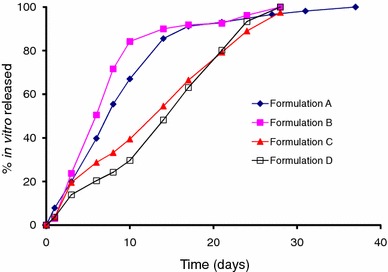
In vitro release of risperidone PLGA microspheres (*Formulations A*, *B*, *C* and *D*)

The in vitro release results for *Formulations A*–*D* are shown in Fig. 1. These results were obtained using the ‘modified dialysis’ method. At first glance, it is evident that *Formulations A and B*, prepared using the 50:50 copolymer, release drug much faster than *Formulations C and D* that were manufactured using a copolymer with a 75:25 lactide:glycolide ratio. With all four formulations, a moderate initial burst was observed (day 1) after which drug release increased to reach between 15 and 20 % by day 3. After this, *Formulations A and B* maintained their release rate such that nearly 50 % of the drug was depleted from the microspheres within a week. In contrast, the release rate for *Formulations C and D* rose in a more sustained fashion to realize 50 % drug release in about 2 weeks, i.e., approximately twice the time observed with formulations containing the 50:50 copolymer. When approximately 85 % drug was released from *Formulations A and B* (i.e., 2 weeks), the drug release rate slowed considerably till complete release was achieved. On the other hand, drug release from the 75:25 copolymer formulations remained consistently steady till 100 % of the drug was released from the microspheres.

Literature cites that drug release from PLGA matrices such as biodegradable microspheres involves three phases (D’Souza et al. 2013a, 2014b). The first phase of release occurs immediately after the microsphere encounters a liquid medium (i.e., in vitro buffer or in vivo fluids). This phase of drug release is termed as ‘initial burst’ and is attributed to the release of drug that is bound to the surface of the microsphere or associated with easily accessible pores. The next two phases of drug release, i.e., diffusional and erosional release, are non-instantaneous and occur over a varying time course. During the diffusional phase of release, water intrusion leads to polymer hydration and slow movement of the encapsulated drug to the outer sink. The presence of water inside the polymer ensures hydrolysis of the polymeric ester bonds causing an autocatalytic effect leading to bulk hydrolysis. Rapid polymer degradation ensues, followed by erosion of the PLGA matrix and mass loss. Thus, drug release rates are much faster during the erosional phase. From Fig. 1, it is evident that all four formulations hydrated rapidly after which drug release rates rose till complete release was achieved.

The differences in vitro behavior of the four formulations can be explained on the basis of the lactide content of these microspheres. Previous studies have documented the differences in the in vitro release profiles between the 50:50 and 75:25 copolymers and ascribed it to a faster degradation rate in copolymers with lower lactide content, i.e., 50:50 PLGA will degrade much faster than the 75:25 copolymer (D’Souza et al. 2014b; Park 1995). Indeed, in comparison to the smaller glycolide species, greater amounts of the larger, more sterically hindered lactide moiety will reduce the degradation rate in a polymer.

Within the same copolymer, a comparison of release profiles for *Formulations A and B* (prepared using 50:50 PLGA) revealed slightly faster release for the higher molecular weight *Formulation B* (Fig. 1). This was presumably due to the higher drug load (i.e., higher drug to polymer ratio) and lower bulk density for *Formulation B* (i.e., 0.67 vs. 0.76 g/cc for *Formulation A*) (D’Souza et al. 2013b). Between *Formulations C and D*, the former exhibited slightly faster drug release that could be attributed to its lower molecular weight.

Overall, the in vitro release experiments revealed the following:

- A moderate initial burst for all formulations;
- Faster drug release from the 50:50 copolymers; and
- Suitability of the ‘modified dialysis’ method in assessing the in vitro release from the four formulations.

### In vivo results

**Fig. 2:**
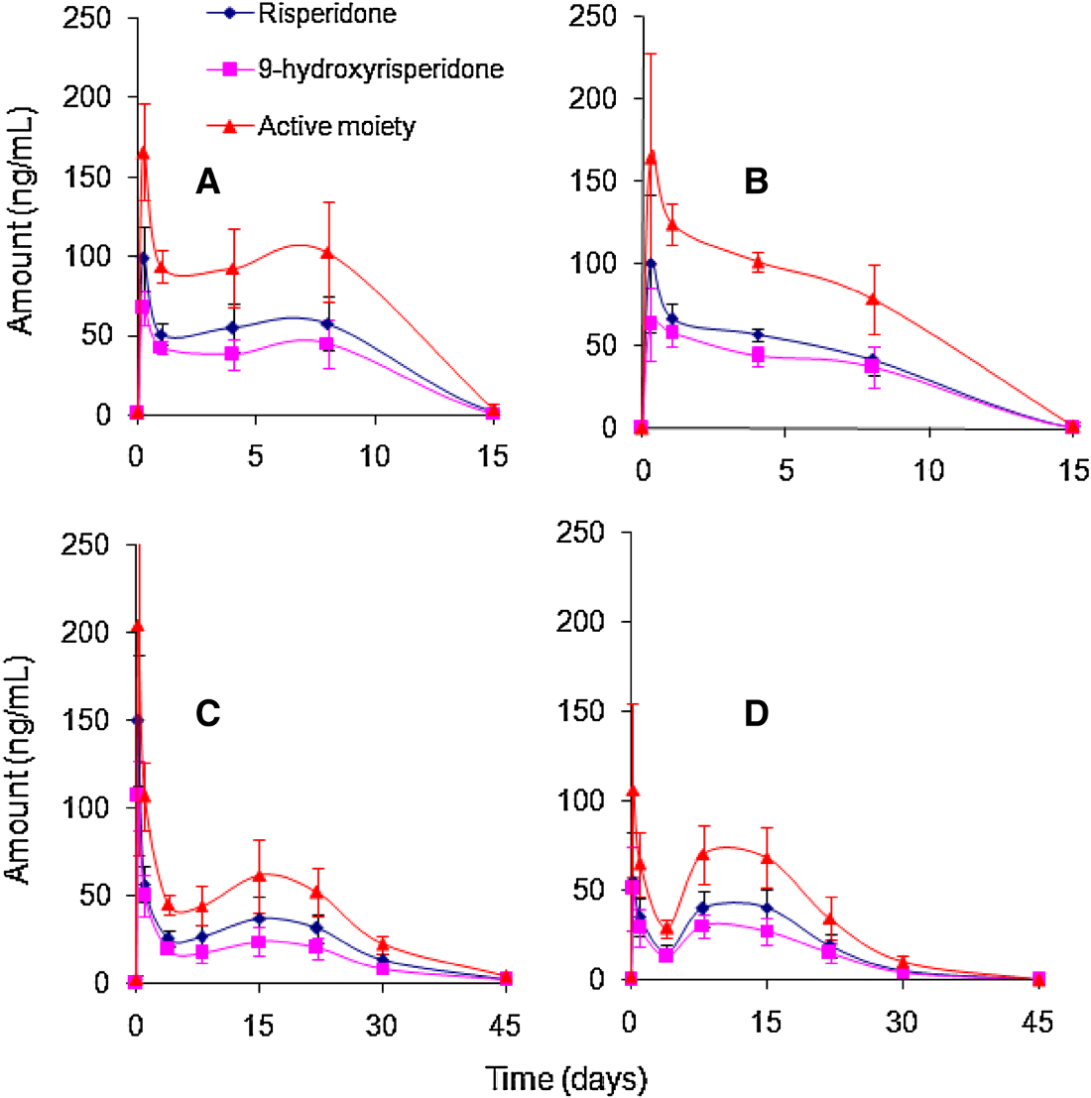
In vivo release of risperidone from PLGA microspheres (*Formulations A* and *B* = 20 mg dose, and *Formulations C* and *D* = 40 mg dose) [data from ref. D’Souza et al. (2013b)]

In a previous study, we reported the preparation, characterization and in vivo evaluation of risperidone PLGA microspheres (D’Souza et al. 2013b). *Formulations A and B* were administered to rats at a 20 mg/kg dose, while *Formulations C and D* were administered at a 40 mg/kg dose. The in vivo release profiles for the four formulations are shown in Fig. 2.

#### Fraction absorbed

**Fig. 3:**
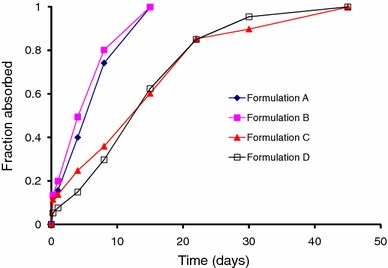
Fraction of risperidone absorbed in vivo (Nelson–Wagner method)

Figure 3 shows the fraction absorbed as a function of time for *Formulations A and B* (20 mg/kg dose) and *Formulations C and D* (40 mg/kg dose) in rats. The fraction of drug absorbed was calculated using the Nelson–Wagner method (described in Sect. “Development of an IVIVC”), i.e., by deconvoluting the pharmacokinetic profile to obtain the in vivo absorption as a function of time (Wagner and Nelson 1963). Deconvolution is a numerical method used to estimate the time course of drug input using a mathematical function. After deconvolution, the in vivo and in vitro release profiles are plotted together to assess the superimposability of the two curves, allowing a direct comparison of release behavior.

A plot of the fraction of drug absorbed for the four formulations is shown in Fig. 3. In a manner similar to the in vitro release profiles described in Fig. 1, the differences in the fraction absorbed for the four formulations are easily discernible and a rank order is assigned. Of the four formulations, *Formulations A*–*C* show a much larger initial burst than *Formulation D*. The initial burst value at day 1 for *Formulation D* was around 7 %, nearly two to three times lower than the 13–20 % drug absorption seen with *Formulations A*–*C*. Between the copolymers, the 50:50 PLGA formulations (*Formulations A and B*) demonstrate a rapid absorption profile, while *Formulations C and D* indicate a more sustained absorption curve. In fact, the absorption profiles including initial burst release for *Formulations A and B* are nearly identical, similar to the in vitro release profiles (Fig. 1) and serum data from rats (Fig. 2) with complete release achieved within 15 days. On the other hand, between the 75:25 polymers, *Formulation D* exhibits slightly slower absorption profile through day 8, after which the rate of absorption increases and is nearly the same as *Formulation C*.

The differences in the fraction of active moiety absorbed from the 50:50 and 75:25 copolymers are clearly evident from day 4 where the value observed from *Formulations A and B* was nearly twice the amount noted with *Formulations C and D*. This trend continued through day 15, when complete absorption was observed from formulations prepared with 50:50 PLGA. In contrast, the time taken for complete absorption for formulations prepared with the 75:25 copolymer was nearly thrice as long as with the 50:50 PLGA. Notably, however, the in vivo drug release profiles obtained by deconvolution (Nelson–Wagner method) were similar, but slightly faster than that observed under in vitro conditions, indicating that the mechanism of release was unchanged.

#### Fractional AUC

**Fig. 4:**
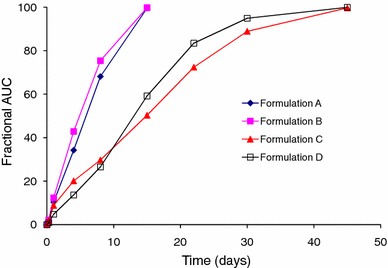
Fractional AUC profile of risperidone from *Formulations A*, *B*, *C* and *D*

Figure 4 depicts a plot of the fractional AUC profile for the four formulations, calculated as described in Sect. “Development of an IVIVC”. In a manner similar to the in vitro release profiles (Fig. 1), the fractional AUC profiles show rank order behavior. Akin to the fraction absorbed (Fig. 3), *Formulation D* had the lowest initial burst, nearly two to three times lower than the remaining formulations (Fig. 4). After the initial burst release of drug, fractional AUC profiles of Formulations *A and B*, prepared using the 50:50 polymer, are essentially similar, with slight differences in the behavior observed between *Formulations C and D*, prepared using the 75:25 polymer. Complete release was achieved in 15 days and 45 days for the 50:50 and 75:25 PLGA formulations, respectively, corroborating the release pattern observed in Fig. 3 (fraction absorbed).

As noted with the in vitro data (Fig. 1) and fraction absorbed (Fig. 3), the microspheres prepared from the lower lactide-containing copolymer exhibited a faster release rate than the high lactide-containing copolymer. The fractional AUC profiles for *Formulations A and B* are nearly identical throughout the time course of drug release. With Formulations C and D, *Formulation D* exhibits slightly slower absorption profile through day 8 after which the rate of absorption increases and is slightly greater than *Formulation C*. Starting from day 4, the differences in fractional AUC profiles between the 50:50 and 75:25 copolymers illustrate that the lower lactide-containing microspheres release drug twice as fast as the higher lactide-containing preparations. This behavior continued till complete release was attained with the 50:50 PLGA preparations. Similar to the profiles shown in Fig. 3 (fraction absorbed), the time taken for complete release with the 75:25 copolymer was threefold greater than the 50:50 PLGA polymer. Overall, the in vivo drug release profiles obtained using the fractional AUC approach were similar to that observed under in vitro conditions, indicating that the mechanism of release was unchanged.

### IVIVC

From the FDA guidance for extended-release oral dosage forms, the most common approach for developing a Level A IVIVC encompasses the steps below:

- Develop formulations with different release rates (e.g., fast, intermediate, slow);
- Obtain in vitro and in vivo release profiles for the formulations; and
- Use an appropriate deconvolution technique (e.g., Nelson–Wagner) to calculate the in vivo absorption after which a correlation may be obtained by comparing in vivo behavior with in vitro release to establish an IVIVC (FDA guidance for industry: extended release oral dosage forms: development, evaluation and application of in vitro/in vivo correlations 1997).

The objective of an IVIVC is to establish a correlation between the in vitro dissolution behavior and in vivo performance of a drug product. Per the guidance, an IVIVC should be demonstrated with two or more formulations of different release rates and show corresponding differences in their absorption profiles. While three or more formulations with different release rates are recommended, an IVIVC may also be defined with a minimum of two or more formulations having different release rates (FDA guidance for industry: extended release oral dosage forms: development, evaluation and application of in vitro/in vivo correlations 1997). As such, an IVIVC is generally described by a linear relationship between parameters derived from the in vitro and in vivo experiments as quantified by the Pearson correlation.

From a drug product perspective, establishing an IVIVC offers several benefits. As noted in literature, having an IVIVC allows in vitro release to be used as a surrogate for in vivo measurements from conventional or long-acting dosage forms (D’Souza and DeLuca 2006). Moreover, it reduces the time, labor and costs associated with performing bio-studies in humans or animals, while also minimizing unnecessary use of humans or animals for evaluation of drug release. For drugs that are in the late stages of development, IVIVCs can play an important role in characterizing process-related changes and also simplify any scale-up or post-approval changes, or to obtain a bio-waiver. Finally, it ensures compliance with regulatory requirements and allows for clinically relevant in vitro dissolution specifications to be set (D’Souza et al. 2014b).

Based on the FDA guidance, a few publications have attempted to investigate an IVIVC from risperidone PLGA systems. For example, Su et al. (2009) investigated the in vivo behavior of several batches of risperidone microspheres formulated using uncapped and capped 75:25 PLGA. Using the ‘sample and separate’ method to assess in vitro drug release, a Level A IVIVC was obtained for a single batch of microspheres. In another publication, Amann et al. (2010) prepared risperidone implants with a series of PLGA polymers and obtained a good Level B correlation with the ‘sample and separate’ method. Given the advantages of the ‘dialysis’ method over the ‘sample and separate’ method, there is a strong rationale for using this technique to establish an IVIVC.

From the current study, *Formulations A and B* had the fastest release rate, while *Formulation Cand D* had a slower release rate. Hence, all four formulations were deemed suitable for analysis of their release profiles with the goal of establishing an IVIVC. As described in Sect. “Development of an IVIVC”, IVIVC was attempted by using two approaches. This type of data analysis wherein IVIVC was attempted and successfully achieved has been reported recently (D’Souza et al. 2014b). The first approach compares the in vitro release profiles to the fractional AUC curves, as reported previously by several authors (Woo et al. 2001; D’Souza et al. 2014b; Chu et al. 2006), while the second approach uses the FDA-recommended Nelson–Wagner method to calculate the absorption profile of the drug (FDA guidance for industry: SUPAC-SS nonsterile semisolid dosage forms. scale-up and post-approval changes: chemistry, manufacturing, and controls; in vitro release testing and in vivo bioequivalence documentation 1997). For analysis using the Nelson–Wagner approach, the fraction absorbed (Fig. 3) was multiplied by 100.

**Fig. 5:**
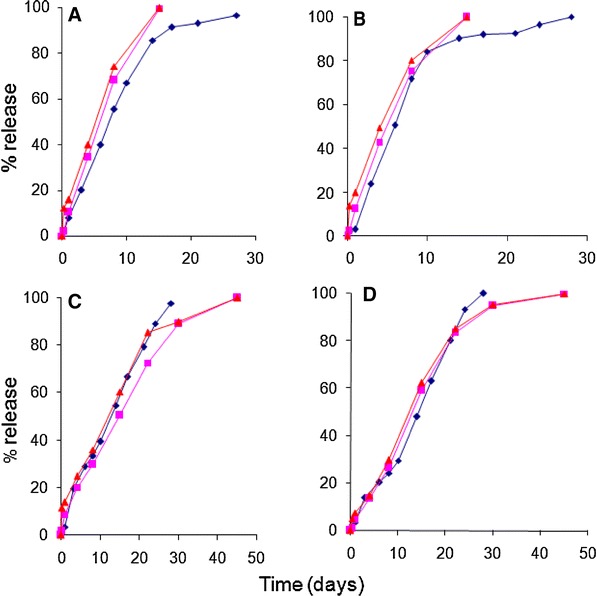
Comparison of in vitro and in vivo release of Risperidone from PLGA microspheres (*diamonds* in vitro release, *squares* fractional AUC, *triangle* Nelson–Wagner absorption)

Figure 5 compares the plots of the fraction absorbed (i.e., *F*_abs_ × 100) and fractional AUC with the in vitro data and highlights few noteworthy findings:

- Near superimposability of the release profiles was obtained in vitro (using the ‘modified dialysis’ method) with in vivo results. Similar findings were reported recently with another atypical antipsychotic, olanzapine, using the ‘modified dialysis’ method (D’Souza et al. 2014b).
- In vivo release, as measured by fractional AUC and fraction absorbed, is slightly faster than in vitro release. This phenomenon has been documented previously and attributed to the contribution of enzymes and foreign body response (Jiang et al. 2003). However, the shapes of the release profiles are essentially indistinct, and the time taken for nearly 90 % drug release is very similar.

**Fig. 6:**
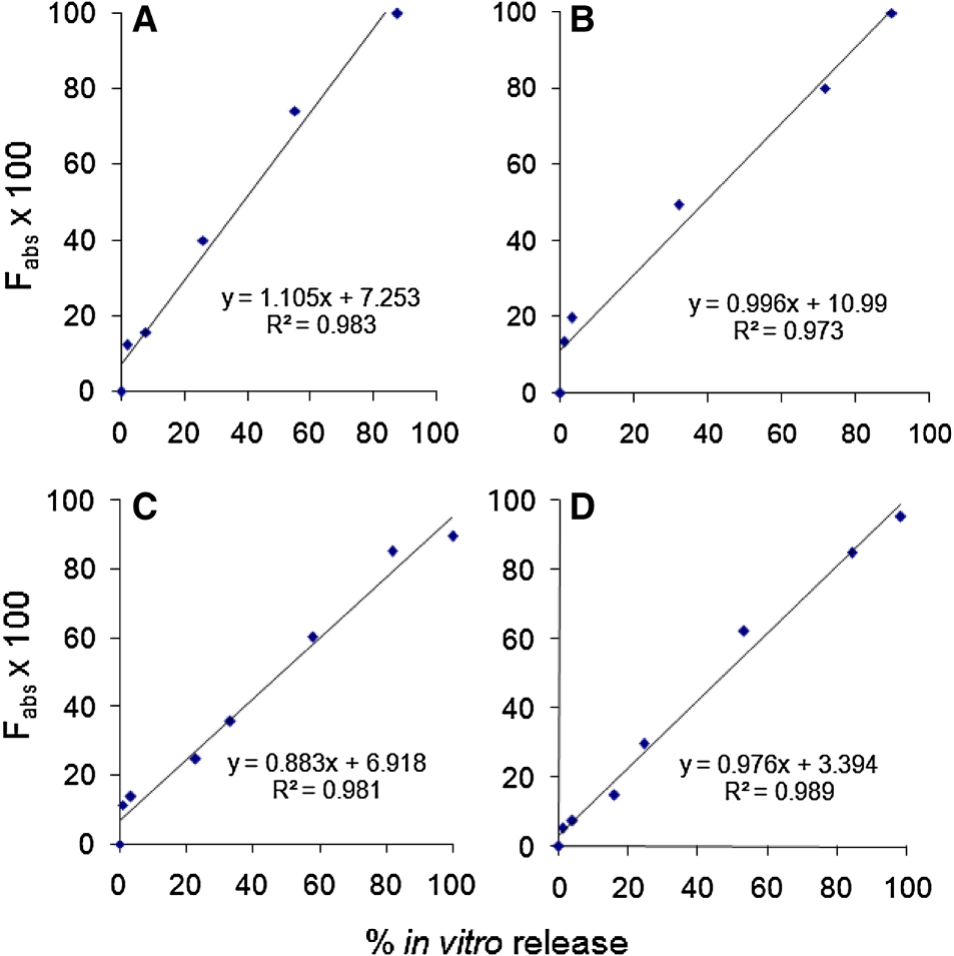
Level A IVIVC for risperidone PLGA microspheres using Nelson–Wagner method

**Fig. 7:**
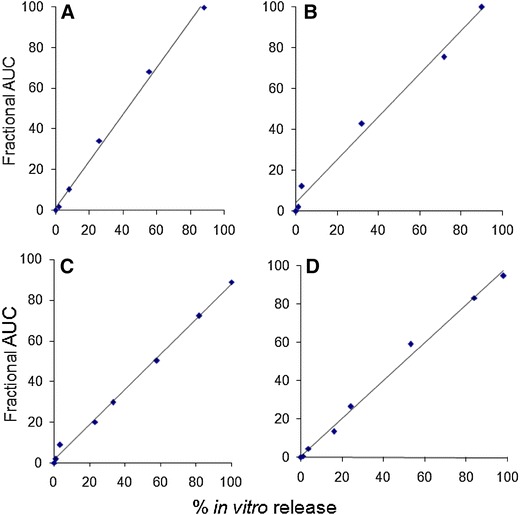
Level A IVIVC for risperidone PLGA microspheres using fractional AUC profile

Figures 6, 7 and Table 1 describe the relationship between % in vitro release using the ‘modified dialysis’ method and the % absorption, as calculated by the Nelson–Wagner method and fractional AUC. As can be clearly seen in the figures and the table, there was an excellent linear correlation (*R*^2^ values between 0.97 and 0.99, *P* > 0.0001) for the 50:50 (fast release) and 75:25 (slow release) formulations. The values of the slope range between 0.97 and 1.147, indicating that in vivo release occurred slightly faster than in vitro release for *Formulations A*, *B and D*, while a slight lag was observed for *Formulation C* (slope = 0.864 − 0.884). The values for the in vivo absorption models shown in Table 1 also attest to the suitability of using either approach to establishing an IVIVC. Indeed, the values obtained for the slope, intercept and *R*^2^ are remarkably similar. Additionally, findings from the current study are in strong agreement with previous data where results from the fractional AUC approach have been comparable to the data generated using the Nelson–Wagner method (Chu et al. 2006; D’Souza et al. 2014b).

**Table 1:** IVIVC fit with in vivo absorption models

Formulation	In vivo absorption model	Slope	Intercept	*R* ^2^
A	Fractional AUC	1.147	1.401	0.996
	Fraction absorbed	1.105	7.253	0.983
B	Fractional AUC	1.050	4.190	0.989
	Fraction absorbed	0.996	10.99	0.974
C	Fractional AUC	0.864	1.534	0.999
	Fraction absorbed	0.884	6.917	0.981
D	Fractional AUC	0.989	0.821	0.995
	Fraction absorbed	0.976	3.395	0.990

**Fig. 8:**
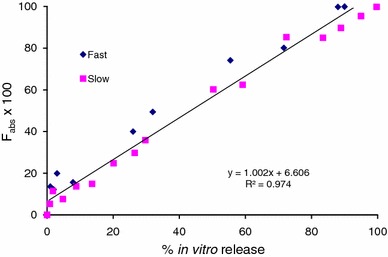
Pooled IVIVC for risperidone PLGA microsphere formulations using Nelson–Wagner method (Level A)

**Fig. 9:**
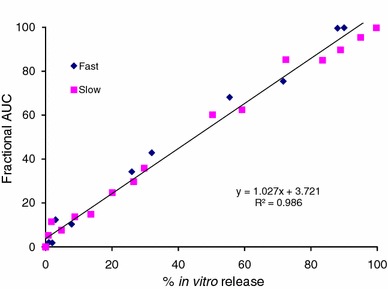
Pooled IVIVC for risperidone PLGA microsphere formulations using fractional AUC profile (Level A)

As recommended by the FDA guidance (FDA guidance for industry: extended-release oral dosage forms: development, evaluation and application of in vitro/in vivo correlations 1997), Figs. 8 and 9 depict the pooled IVIVCs for the 50:50 (fast release) and 75:25 (slow release) risperidone PLGA formulations. The results show an excellent Level A correlation between the in vitro data, obtained using the ‘modified dialysis’ method, and the in vivo results, using the fraction absorbed (Fig. 8) and the fractional AUC (Fig. 9) profiles. With both analyses, values of the slope were nearly equal to 1, confirming that in vivo release for the slow and fast release formulations (50:50 and 75:25 PLGA) followed a similar trend, with minimal lag. The intercept values ranged between 3 and 6; with a higher value obtained with the Nelson–Wagner approach, as described in Sect. “Fraction absorbed”. In summary, the results in Figs. 8 and 9 demonstrate an excellent Level A correlation (*R*^2^ value greater than 0.97) between in vitro release of risperidone from PLGA microspheres and in vivo release. From literature, such type of correlation has not been previously reported with this molecule (Amann et al. 2010; Su et al. 2009).

While reports have cited the lack of standardized in vitro test methods as an important reason for dearth of an IVIVC or a lack of 1:1 correlation with parenteral microspheres (Rawat et al. 2012), data from the current study prove that with proper selection of an in vitro method, an IVIVC can be obtained. A similar finding and a near 1:1 correlation was also recently reported on olanzapine PLGA microspheres by our group (D’Souza et al. 2014b). Thus, results from the current study indicate that in vitro release using the ‘modified dialysis’ method is an excellent predictor of in vivo behavior of small molecules such as risperidone encapsulated into PLGA matrices and can be used as an indirect measure or surrogate for in vivo performance from developmental or clinical dosage forms.

## Conclusions

A ‘modified dialysis’ method was selected to evaluate the in vitro behavior of risperidone PLGA microspheres formulated using 50:50 and 75:25 PLGA copolymers. This method was discriminatory and able to accurately distinguish the formulations on the basis of their release rates. In vitro release profiles exhibited a rank order similar to the results obtained in vivo by the FDA-recommended deconvolution approach (Nelson–Wagner method) or fractional AUC. Using both in vivo approaches, a near 1:1 linear Level A correlation between in vitro and in vivo release was obtained for the four formulations evaluated, suggesting that the ‘modified dialysis’ technique was suitable for in vitro release assessment of risperidone PLGA dosage forms.
